# Investigating the Potential of X-Ray-Based Cancer Treatment Equipment for the Sterile Insect Technique in *Aedes aegypti* Control Programs

**DOI:** 10.3390/insects15110898

**Published:** 2024-11-18

**Authors:** Linmin Wang, Tingting Liu, Liang Xiao, Haiting Zhang, Cunchen Wang, Weixian Zhang, Mao Zhang, Ying Wang, Shengqun Deng

**Affiliations:** 1 Department of Pathogen Biology, Anhui Province Key Laboratory of Zoonoses, The Provincial Key Laboratory of Zoonoses of High Institutions in Anhui, School of Basic Medical Sciences, Anhui Medical University, Hefei 230032, China; 2245010044@stu.ahmu.edu.cn (L.W.); zhanghaiting0919@163.com (H.Z.); 17861282695@163.com (C.W.); 15732652137@163.com (W.Z.); 2 Department of Tropical Medicine, College of Military Preventive Medicine, Army Medical University, Chongqing 400038, China; xinting0627@126.com; 3 Department of Radiotherapy, The First Affiliated Hospital of Anhui Medical University, Hefei 230022, China; xiaoliang@ahmu.edu.cn

**Keywords:** mosquito control, radiotherapy equipment, sterile insect technique, *Aedes aegypti*

## Abstract

The sterile insect technique (SIT) stands as an eco-friendly approach for mosquito control, but it is impeded by the limited availability of γ-ray radiation source. This research sought to investigate a different radiation source—the Varian Clinac 23EX linear accelerator, which is frequently used for X-ray therapy in cancer treatment. Evaluation parameters including emergence rate, average survival time, induced sterility (IS), male mating competitiveness of irradiated males and fecundity (the number of eggs per female per batch), and the egg hatch rate of females mated with irradiated males were assessed to gauge the application potential of this cancer treatment equipment in the realm of the SIT. The results indicated that X-rays from radiation therapy equipment can effectively suppress the hatch rate of offspring mosquitoes without adversely affecting the emergence rate of irradiated males or the fecundity of females. In addition, at an X-ray dose of 60 Gy, the induced sterility in *Ae. aegypti* was comparable to the sterility induced by 40 Gy of γ-rays with both treatments resulting in 99.6% sterility. Interestingly, when a release ratio of 7:1 (irradiated males:unirradiated males) was used to competitively mate with females, the IS results resulted by 60 Gy X-rays and 40 Gy γ-rays were still at 70.3% and 73.7%, respectively. In conclusion, the results underscored the potential of the Varian Clinac 23EX linear accelerator as an X-ray source in SIT research.

## Simple Summary

Our research delved into the potential of using the Varian Clinac 23EX linear accelerator, typically employed in X-ray cancer treatment, as an alternative radiation source for the sterile insect technique (SIT) in mosquito control. Through our investigation, we found that by adjusting the X-ray dosage to a specific threshold, the resulting sterilization effect rivaled that achieved by traditional γ-ray sources. For example, at a dose of 60 Gy of X-rays and 40 Gy of γ-rays, the egg hatch rate can even be as low as 0.3%, which can result in induced sterility (IS) of 99.6%. This breakthrough opens up a convenient avenue for researchers, granting them access to readily available radiotherapy equipment for SIT studies. By leveraging the existing infrastructure, such as radiotherapy facilities, researchers can expedite their SIT research endeavors, potentially revolutionizing mosquito control efforts. The integration of radiotherapy equipment into SIT studies promises to propel advancements in this field, facilitating faster progress and fostering eco-friendly approaches to insect population management.

## 1. Introduction

*Aedes* mosquitoes are important vectors of arboviral diseases, such as dengue, Zika, and chikungunya, posing an enormous health threat to human [1,2]. Over the past 30 years, for example, the dengue virus has caused an increasing global burden, especially in recent years, when it caused more than 390 million infections per year [3,4]. Moreover, in the past few decades, the population and distribution of *Aedes* mosquitoes have increased significantly [5,6]. Traditional mosquito control methods mainly rely on chemical insecticides and the removal of mosquito breeding sites, but these two methods have limited scope and success rates in controlling mosquito populations [7]. While chemical insecticides can be effective, they also pose risks to non-target organisms, leading to environmental concerns. Furthermore, the overuse of these chemicals can result in increased resistance among mosquito populations, making them less effective over time [8]. In addition, it is often challenging to completely eliminate mosquito breeding. As a result, there is an immediate need to investigate new eco-friendly approaches for managing mosquito populations.

The sterile insect technique (SIT), which utilizes radiation, effectively decreases the population density of target mosquitoes by introducing a considerable number of sterile male mosquitoes that mate with wild females [9,10]. This method serves as an autocidal biological control approach [11]. As a key component of comprehensive area-wide pest management strategies, the SIT predominantly employs γ-rays from isotopic sources [12]. The International Atomic Energy Agency (IAEA) has stricter control over cobalt-60 radiation sources for safety reasons. In addition, the price to recharge a gamma cell is very expensive compared to the price of a new X-ray machine [13]. The improper disposal of gamma source after use will also cause environmental pollution [14]. In contrast, X-rays are released discontinuously and do not produce radioactive waste. In the past decade, many studies have shown that X-rays can be used as a radiation source for the SIT [11,15,16,17]. For example, the codling moth *Cydia pomonella* was 100% sterile after X-ray irradiation of 366 Gy or higher [18]. Research by Yamada et al. [19] has shown that blood X-ray irradiators can be used as irradiators for the SIT to irradiate insects such as fruit flies, tsetse flies, and mosquitoes. Zhang et al. [20] have demonstrated that X-rays can induce chromosomal damage in the sperm of male *Aedes aegypti* mosquitoes, resulting in male sterility. Given the availability of various radiation sources for X-ray exposure, our focus has been on utilizing the commonly used X-ray tumor radiotherapy equipment found in hospitals for this purpose to ensure more convenient access to radiation sources.

Malignant tumors have emerged as a significant global health threat to humans [21]. Radiation therapy is a key approach in cancer treatment, utilizing high-energy radiation to hinder or reduce the proliferation of tumor cells [22]. Due to the increasing demand for cancer treatment, X-ray therapy equipment has enormous market potential in China [23,24]. Given this, researchers can irradiate at the nearest hospital’s radiology department to reduce the difficulty of finding radiation sources and avoid harm to mosquitoes during transportation [25]. In addition, collaborating with hospitals to conduct SIT experiments using radiotherapy equipment during off-peak hours can reduce a significant amount of experimental costs for researchers. However, there are still many research issues on sterility male mosquitoes that need to be solved before the application of X-rays in the SIT.

Linear accelerator radiotherapy systems are commonly used devices for treating malignant tumors, with the advantages of targeting tumors and high-precision avoidance of normal tissues [26]. Varian Medical Systems have a long and rich history as pioneers in the oncology field. The Varian Clinac linear accelerator, which has demonstrated effective therapeutic outcomes in the treatment of breast cancer, prostate cancer, meningioma, lung cancer, melanoma, and total skin electron therapy, was employed in our investigation [27,28,29,30,31,32]. In 2017, researchers showed that the Varian Clinac 21EX linear accelerator can utilize ultrahigh dose rates for mouse irradiation and achieved excellent dosimetric properties at doses exceeding 200 Gy/s [33]. The Varian Clinac 23EX linear accelerator located at the Department of Radiotherapy, the First Affiliated Hospital of Anhui Medical University, Hefei, China, was used in this study as a radiation source for X-rays to irradiate the male pupae of *Ae. aegypti* mosquitoes due to its convenience and accessibility. This Varian Clinac 23EX linear accelerator can offer two options for photon beams: −6 MV and 18 MV. Its operating platform enables precise and reproducible positioning, along with dynamic control and timely beam opening, to minimize radiation exposure.

Our experiment primarily utilized therapy equipment to deliver X-rays to *Ae. aegypti* mosquitoes and evaluated their effectiveness in the SIT compared to traditional γ-rays. This evaluation involved the examination of the emergence rate, the survival time of irradiated males, the number of eggs per unirradiated female, and the egg hatch rate after mating with irradiated males of *Ae. aegypti* mosquitoes. Furthermore, we conducted tests on male mating competitiveness and induced sterility (IS) in *Ae. aegypti* mosquitoes under different ratios of irradiated males:unirradiated males (release ratio).

## 2. Materials and Methods

### 2.1. Mosquitoes

*Aedes aegypti* mosquitoes from Zhanjiang City, originally obtained from the Guangdong Provincial Center for Disease Control and Prevention in China, have been cultured in our laboratory since 2020. These mosquitoes were maintained in an environment with controlled conditions: a temperature range of 28 ± 1°C, humidity levels at 80 ± 5%, and a photoperiod of 14 h of light and 10 h of darkness. Mosquito larvae were fed with turtle food (sourced from Shenzhen INCH-GOLD Fish Food, LTD, Shenzhen, China) daily, while adult mosquitoes received a 10% glucose solution as their source of nutrition. Following mating, female mosquitoes were offered Kunming mice (supplied by the Animal Experiment Center of Anhui Medical University) for blood feeding. Subsequently, we placed a plastic bowl containing moist filter paper inside the mosquito cage to collect eggs. All animal procedures were conducted in accordance with the guidelines approved by the Experimental Animal Ethics Committee of Anhui Medical University (approval code: LLSC20210773).

### 2.2. Radiation Equipment

A cobalt-60 irradiation device at the Institute of Technical Biology and Agriculture Engineering, Hefei Institutes of Physical Science, Chinese Academy of Science, Hefei, China, supplied the γ radiation sources. The Varian Clinac 23EX linear accelerator (Varian, Palo Alto, CA, USA) from the Department of Radiotherapy, the First Affiliated Hospital of Anhui Medical University, Hefei, China, was used for X-irradiation.

The cobalt-60 γ-ray radiation source powers a 1.5 MeV electron irradiation test device primarily used for intermittent testing of small-sized products. The energy can be continuously adjusted from 1.0 to 1.5 MeV, while the beam current is adjustable from 0.5 to 6.0 mA. By integrating multiple under-beam devices, both static and dynamic irradiation modes can be achieved. To ensure dose uniformity, the radiation sources are arranged in a cylindrical configuration, creating an equal dose surface-shaped like a ring.

The Varian Clinac 23EX linear accelerator is equipped with two independent dose monitoring systems that ensure real-time monitoring of dose information, enhancing system accuracy and safety. These independent dose-rate channels can terminate the preset outlet and abort the process if the two channels deviate by more than 10%. Real-time monitoring of the symmetry and uniformity of the dose rate is conducted, with radial angle and position deflection signals transmitted to the console computer. This allows for the limitation, control, and display of symmetrical uniform information regarding the radial rays. If the radial symmetry uniformity data exceeds the set range, the outgoing line is terminated.

### 2.3. Pupal Irradiation

**Figure 1:**
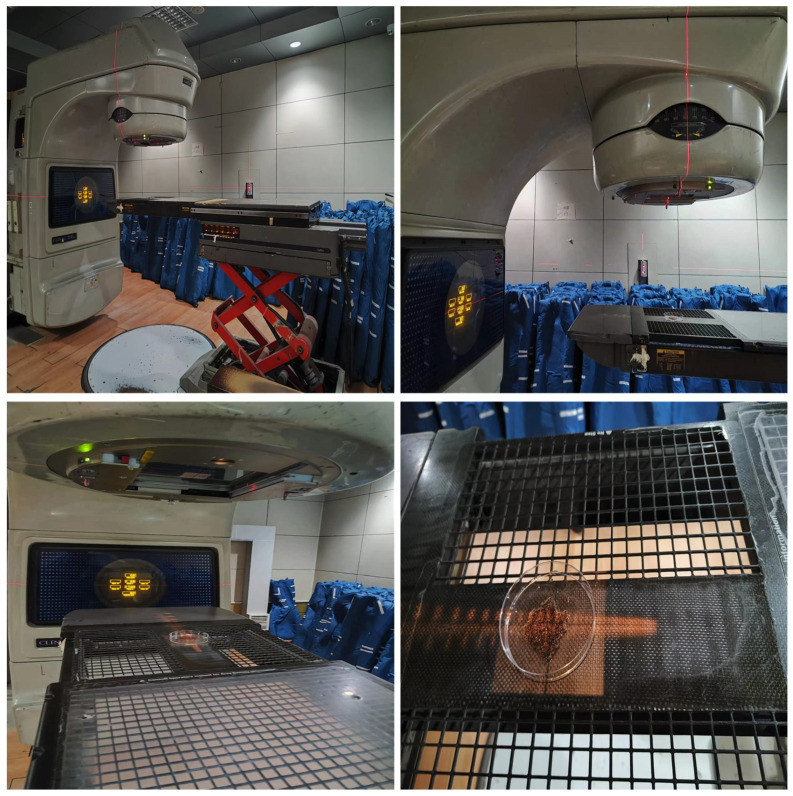
The male pupae of *Aedes aegypti* mosquitoes were irradiated by the Varian Clinac 23EX linear accelerator. The picture of the equipment of Varian Clinac 23EX linear accelerator and the radiation process images (front view, side view, and top view) can be observed.

*Aedes aegypti* from the same batch of eggs were divided into two groups. One group was used to emergence into adult mosquitoes as the control group, while pupae were collected from the other group for irradiation. Approximately 150 to 200 male pupae (lighter colored and smaller sized) aged 12 to 24 h were chosen for exposure to various X-ray and γ-ray doses (20, 40, and 60 Gy). During the X-ray and γ-ray irradiation process, male pupae were first transported in water and subsequently transferred to petri dishes (9 cm in diameter). To ensure uniform dosing, water was removed to eliminate any oxygen interference in the experiments [34]. The pupae were irradiated at a sample depth of 1.5 cm and at a source-surface distance of 100 cm. Once the irradiation was completed, pupae were quickly transferred to water (Figure 1) and placed in new mosquito cages (25 cm × 35 cm × 25 cm) to facilitate their emergence, after which they were paired with virgin females for mating.

### 2.4. The Emergence Rate and Average Survival Time of Irradiated Males

Thirty irradiated male pupae were placed in mosquito cages (dimensions: 25 cm × 35 cm × 25 cm). Two days later, we counted the number of mosquitoes that had emerged in each mosquito cage. The control group consisted of unirradiated male pupae and underwent the same treatment as the experimental group, including transportation. Each experiment was repeated twice, with three parallel groups of 30 irradiated male mosquitoes in each group.

Except for the pupae whose emergence rate was recorded, the remaining irradiated pupae were also housed in mosquito cages for emergence. Subsequently, the emerged males were transferred to fresh cages for further observation. The daily count of surviving male mosquitoes was conducted until the last individual expired. The control group consisted of unirradiated mosquitoes. Each experiment was repeated twice, with three parallel groups of 30 irradiated male mosquitoes in each group.

### 2.5. The Fecundity and Egg Hatch Rate of Females That Mated with Irradiated Males

Males of *Ae. aegypti* that emerged after the exposure to different irradiation doses were placed in separate mosquito cages with unirradiated virgin females of the same strain in a 1:1 ratio (30 males and 30 females). The females were allowed to mate for two days. Subsequently, the females were provided with Kunming mouse blood. Each engorged mosquito was then placed separately in a 9 ounce cup with funnel-shaped filter paper, and then water was added to the filter paper after two days, keeping the filter paper moist to provide a suitable egg laying environment for female mosquitoes. The females were allowed to lay eggs for five days, after which the eggs on each paper were counted using a stereoscope. The control group was the number of eggs laid by each female after mating with unirradiated males. Filter papers containing eggs were dried for subsequent experiments. With 100 eggs in a group, the larvae hatched in each group for 5 days were counted, and then the egg hatch rates at different X-ray and γ-ray doses and for the control group were calculated. Each experiment was repeated twice, with three parallel groups of 30 irradiated male mosquitoes in each group. The following equations were utilized to determine the IS [35] in each group:

IS = (1 − Hc/Hn) × 100%

In this equation, Hc denotes the hatch rate of each group, and Hn indicates the hatch rate of the control group (0 Gy).

### 2.6. The Male Mating Competitiveness and Induced Sterility at Different Release Ratios

Based on the findings from the previous experiments, we selected 60 Gy of X-rays and 40 Gy of γ-rays to irradiate male *Ae. aegypti* pupae. Male pupae aged 12 to 24 h were exposed to 60 Gy of X-rays and 40 Gy of γ-rays and subsequently moved to new mosquito cages. In these cages, we introduced varying numbers of irradiated males—30, 30, 90, 150, 210, and 0—paired with 0, 30, 30, 30, 30, and 30 unirradiated males, respectively, to set the release ratios at 1:0, 1:1, 3:1, 5:1, 7:1, and 0:1 (control). Each cage housed 30 unirradiated virgin females. After a 3-day period of mating competition, the females were provided with blood meals. Following this, each engorged mosquito was then separately placed in a 9 ounce cup with funnel-shaped filter paper, and then water was added to the filter paper after two days, keeping the filter paper moist to provide a suitable egg laying environment for female mosquitoes. The total number of eggs on each paper for 5 days was recorded, followed by a 1 week incubation period. Each experimental setup was conducted twice, with three replicates per treatment group. The following equations were utilized to determine the male mating competition index (*C*) [36] at different release ratios:

*C* = (Hn − Hc)/(Hc − Hs) × N/S

In this equation, Hc is the hatch rate of the competition group (which includes ratios of 1:0, 1:1, 3:1, 5:1, 7:1, and 0:1 for irradiated to unirradiated males); Hn is the hatch rate of the negative control group (the release ratio of 0:1 for irradiated to unirradiated males); Hs represents the hatch rate of the irradiated positive control group (the release ratio of 1:0 for irradiated to unirradiated males); N is the count of unirradiated males; and S is the count of irradiated males.

### 2.7. Statistical Analysis

IBM SPSS version 20 was used for statistical analysis of all data in this paper. To compare differences in emergence rate and egg hatch rate among groups, Pearson Chi-square tests and Bonferroni adjustments were employed. Kaplan–Meier analysis along with log-rank (Mantel–Cox) tests were utilized to assess survival differences among the groups. The number of eggs, induced sterility, and male mating competitiveness were analyzed using ANOVA with Tukey post hoc tests. All data are expressed as mean ± standard error of the mean (SEM), and values with *p* < 0.05 were deemed statistically significant.

## 3. Results

### 3.1. The Male Emergence Rate Subsequent to Radiation Exposure

**Figure 2:**
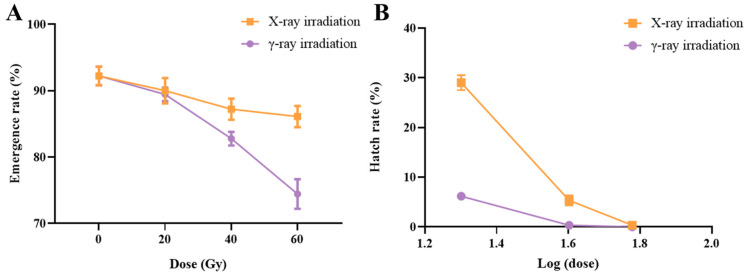
Effects of X-ray and γ-ray irradiation on the emergence rate (**A**) and egg hatch rate (**B**) of *Aedes aegypti*. The error bar indicates ± SEM. (Pearson chi-square and Bonferroni tests.)

The successful emergence of irradiated males is the first step in their effectiveness. For *Ae. aegypti*, compared with the control group, there was no significant difference (χ2 = 4.163, df = 3, *p* = 0.244) in the emergence rate at any dose of X-rays; even for 60 Gy of X-rays, the emergence rate remained at 86.1 ± 1.2% (Figure 2A, Table 1). When the γ-ray dose was 20 Gy, there was no significant difference in emergence rates compared to the control group (χ2 = 0.834, df = 1, *p* = 0.361). However, at a γ-ray dose of 60 Gy, the emergence rate was and 74.4 ± 1.6%, which was significantly lower than that of both the control group and the equivalent dose of X-rays (Figure 2A, Table 1, control group: χ2 = 20.480, df = 1, *p* < 0.001; 60 Gy of X-rays: χ2 = 7.737, df = 1, *p* = 0.005).

### 3.2. The Fecundity, Egg Hatch Rate, and Induced Sterility of Irradiated Males

We evaluated the fecundity (number of eggs per female per batch) and egg hatch rate of unirradiated virgin females mated with males irradiated at different doses of X-rays and γ-rays. The IS of males at different doses of X-rays and γ-rays were determined using the respective hatch rates. Interestingly, none of the X- or γ-ray doses affected fecundity (X-rays: F = 0.266, df = 3, *p* = 0.85; γ-rays: F = 0.926, df = 3, *p* = 0.427) (Table 1). However, with the increase in the doses of X-rays and γ-rays, the egg hatch rate of mosquitoes gradually decreased (X-rays: χ2 = 210.9, df = 3, *p* < 0.001; γ-rays: χ2 = 287.373, df = 3, *p* < 0.001) (Figure 2B). At the doses of 20 Gy and 40 Gy, the hatch rate of eggs irradiated using X-rays was significantly higher than that of the same dose of γ-rays (Table 1, 20 Gy: χ2 = 18.32, df = 1, *p* < 0.001; 40 Gy: χ2 = 5.128, df = 1, *p* = 0.024). For example, with a dose of 40 Gy, the egg hatch rates after irradiation with X-rays and γ-rays were 5.3% and 0.3%, respectively (Table 1). At a dose of 60 Gy, the egg hatch rate by X-rays and γ-rays were as low as 0.3% and 0%, respectively, which resulted in IS of 99.6% and 100% (χ2 = 0, df = 1, *p* = 1). Substantially, a higher dose of X-rays could achieve a sterility effect comparable to that of a lower dose of γ-rays.

### 3.3. The Average Survival Time of Irradiated Males

**Figure 3:**
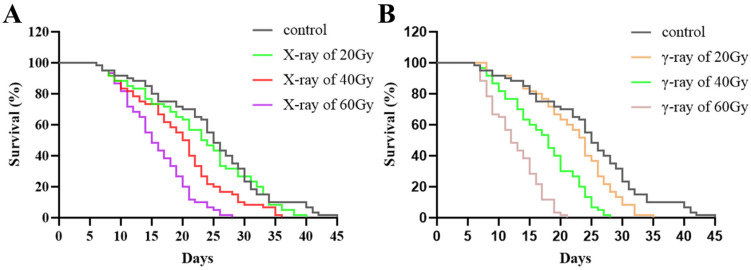
Survival curves of *Aedes aegypti* males irradiated with different doses of X-rays (**A**) and γ-rays (**B**).

The extended survival duration of irradiated male post-emergence enhances their chances of successfully mating with females. We assessed the survival times of males subjected to various doses of X-rays and γ-rays. In the case of *Ae. aegypti*, radiation significantly decreased the average survival time. As the dose increased, the reduction in the survival time became more pronounced (Figure 3). The impact of γ-rays on the average survival time of males was significantly greater compared to that of X-rays (Table 2, 20 Gy: χ2 = 245.115, df = 1, *p* < 0.001; 40 Gy: χ2 = 176.899, df = 1, *p* < 0.001; 60 Gy: χ2 = 227.581, df = 1, *p* < 0.001). However, males exposed to γ-rays at 20 Gy had an average survival time of 24.7 ± 0.2 days, comparable to that of males exposed to X-rays at 40 Gy (χ2 = 0.427, df = 6, *p* = 0.514) (Table 2). Notably, at a dose of 60 Gy, the average survival times for males exposed to X-rays and γ-rays were 18.1 ± 0.2 days and 14.3 ± 0.1 days, respectively, both significantly lower than the control group’s average of 29.0 ± 0.2 days (X-rays: χ2 = 1154.085, df = 6, *p* < 0.001; γ-rays: χ2 = 1833.332, df = 6, *p* < 0.001). Our results indicated that radiation can impact the survival time of males, but even under the highest dose of 60 Gy X-ray radiation used during the experiment, males can have an average survival time of two weeks, which will allow them sufficient time to find the wild females and mate with them.

### 3.4. Male Mating Competitiveness and Induced Sterility Under Different Release Ratios of Irradiated Males

Based on the experimental findings on the survival time and egg hatch rate, we selected 60 Gy of X-rays and 40 Gy of γ-rays for the subsequent experiments, as these conditions were most effective in reducing the egg hatch rate. As the release ratios of irradiated males increased, there was a corresponding gradual decrease in the egg hatch rate (X-rays: χ2 = 169.543, df = 5, *p* < 0.001; γ-rays: χ2 = 193.273, df = 5, *p* < 0.001) (Table 3).

**Figure 4:**
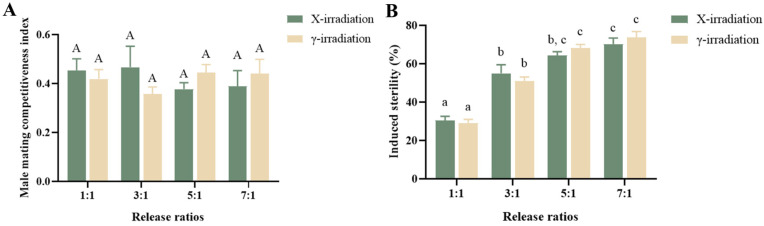
Effects of release ratios on the male mating competitiveness (**A**) and induced sterility (**B**) of *Aedes aegypti* males. The error bar indicates SEM. Values that are accompanied by different letters (A and a–c) indicate significant differences between them, as determined by ANOVA and Tukey’s post hoc tests; *p* < 0.05.

After 60 Gy of X-rays and 40 Gy of γ-rays irradiation, the male mating competitiveness was *C* = 0.45 ± 0.12, and there was no statistically significant difference in *C* at any different release ratios (Figure 4A, F = 0.364, df = 7, *p* = 0.906).

The IS increased progressively with higher release ratios, reaching the highest of 70.3% and 73.7% when the release ratio of males irradiated by 60 Gy of X-rays and 40 Gy of γ-rays, respectively, was 7:1 (Figure 4B). Notably, the egg hatch rates resulted from the mating of males irradiated with the same release ratios of X-rays and γ-rays showed no significant difference (Table 3, different release ratios of irradiated males and unirradiated males: 0:1: χ2 = 0.344, df = 1, *p* = 0.558; 1:1: χ2 = 0.187, df = 1, *p* = 0.666; 3:1: χ2 = 0.523, df = 1, *p* = 0.47; 5:1: χ2 = 0.221, df = 1, *p* = 0.638; 7:1: χ2 = 0.109, df = 1, *p* = 0.741; 1:0: χ2 = 1.005, df = 1, *p* = 0.316).

## 4. Discussion

Radiation damages DNA in living cells of insects, while radiation-induced mutation in germ cells or cells that are under constant division is more serious, which can lead to low sterility or pathological damage of offspring [37,38]. The SIT mainly uses radiation to make male mosquitoes infertile, thereby achieving the goal of suppressing mosquito populations. Cesium-137 and cobalt-60 usually act as radiation sources in the SIT. However, the serious hazards caused by these two radiation sources and their potential high risks, such as the use of cesium-137 as a component of bombs by terrorist organizations, have raised concerns [39]. Linear accelerators mainly emit electrons from an electron gun, which are accelerated by an accelerator tube to high energies. The high-speed electrons collide with a tungsten metal target, producing X-rays [40]. Compared to γ-rays, X-ray sources are easily accessible and cause less radiation pollution [41,42]. In the context of increasingly strict control over γ-ray sources, X-rays are encouraged as an alternative source of radiation for the SIT. With the transition from γ-rays to X-rays as the main source of the SIT ionizing radiation, more comparative studies between the new and old radiation sources are crucial.

The key to using the SIT to control mosquito populations is to release sufficient numbers of sterile males, and these sterile males can successfully compete with wild males for females [43]. This requires that the survival time, male mating competitiveness, and induced sterility of irradiated male mosquitoes should be as close to or even slightly better than those of wild male mosquitoes as possible [39,44]. Designing an electron beam accelerator with X-ray converter specifically for the SIT is too complex and expensive, so we focus our interest on existing linear accelerators [45]. Kim et al. [46] used a high-energy linear accelerator to irradiate *Drosophila suzukii* and found that irradiating pupae at 73 Gy can lead to F1 generation sterility. Chang et al. [47] used a linear electron accelerator to irradiate the pupae of the oriental fruit fly, *Bactrocera dorsalis*, and found that exposure to X-rays can affect the expression of 31 proteins in adult males, resulting in male sterility. The study by Balestrino et al. [48] also indicates that the linear electron accelerator can be used for *Ae. japonicus* control in the SIT.

A linear accelerator from the Radiology Department of the First Affiliated Hospital of Anhui Medical University, the Varian Clinac 23EX linear accelerator, was chosen as our radiation source due to its convenience and accessibility. The Varian medical linear accelerator produces high-energy X-rays and has been widely used for patient cancer radiotherapy [49]. The male pupae of *Ae. aegypti* were exposed to X-rays and compared with traditional γ-irradiation to further explore whether radiation equipment can serve as a radiation source for the SIT in the future.

Encouragingly, our results indicated that 60 Gy of X-rays can achieve a 99.6% sterility effect without affecting the emergence of males. Roselli et al. [50] used a medical linear accelerator from the nearby hospital’s Radiology and Oncology Department to irradiate the brown marmorated stink bug, *Halyomorpha halys*. Unlike us, they used 32 Gy of X-rays of a medical linear accelerator to irradiate male adults, reducing the hatch rate of eggs from females mated to irradiated males to less than 5% without affecting their lifespan or male mating competitiveness. Although the average survival time for males exposed to 60 Gy of X-ray irradiation was 18.1 ± 0.2 days, significantly lower than that of the control group, this drawback can be mitigated by regularly releasing a substantial number of irradiated males [9]. Yamada et al. [11] found that the average survival time of *Ae. albopictus* males subjected to 60 Gy of X-rays was roughly half that of the control group, although their mosquitoes had a longer average lifespan than those in our study. Additionally, our findings indicated that X-rays were less detrimental to the pupae of *Ae. aegypti* compared to the same dose of γ-rays. Males treated with X-rays exhibited longer survival compared to those exposed to the same dosage of γ-rays; however, X-rays were less effective in inducing sterility. Nonetheless, Yamada et al. [19] reported that using the Raycell MK2 Blood X-ray Irradiator, a 55 Gy exposure could achieve 99.9% sterility in *Ae. aegypti*, which was similar to our results. In the experiment conducted by Du et al. [51], the IS in sterile male *Ae. albopictus* was 74% at a release ratio of 7:1. Similarly, in our study with *Ae. aegypti* at the same release ratio of 7:1, the IS results for X-rays and γ-rays were 70.3% and 73.7%, respectively, with no statistically significant difference between the two.

Although we have shown that X-rays with radiotherapy equipment as a radiation source can replace γ-rays in terms of sterility effects, our experiment still has many drawbacks. For example, the ability of an insect to perform flight is a direct marker of mosquito quality, and male mosquitoes with weak flight ability are unable to find females and successfully mate [52]. Therefore, we need to continue conducting field experiments to test the impact of radiation from radiotherapy equipment on the flight ability of male mosquitoes in the future. Zhang et al.’s [20] research indicates that male *Ae. aegypti* mosquitoes infected with *Wolbachia* exhibit increased sensitivity to radiation, achieving complete male sterility under low-dose X-ray exposure (20 to 25 Gy). Combining the SIT using radiation therapy equipment as a radiation source with insect incompatibility techniques and genetically modified mosquitoes may achieve unexpected results, which will also be one of the focuses of our future research [53,54,55,56].

In addition, the Varian Clinac 23EX linear accelerator requires highly trained personnel for its operation and maintenance. This emphasizes the need for skilled professionals to ensure proper functioning. Furthermore, given the high cost of tumor radiotherapy equipment and the challenges of borrowing such equipment for radiation during limited leisure time, the existing machines may serve as an initial solution for SIT projects, but they should not be considered a long-term fix.

## 5. Conclusions

Based on our research findings, it can be concluded that radiotherapy equipment Varian Clinac 23EX linear accelerator demonstrates suitability for basic research on the sterile insect technique (SIT). Our study revealed that by increasing the dose of X-rays, the resulting sterility effect is comparable to that achieved with γ-rays. Increasing the release ratio of sterile male mosquitoes can better induce a decrease in the population of mosquitoes in the wild. Utilizing radiotherapy equipment will provide researchers in the field with convenient access to radiation sources. Furthermore, the incorporation of radiotherapy equipment is expected to greatly accelerate the advancements and progress of SIT research.

## Figures and Tables

**Table 1:** Effects of X-rays and γ-rays on the emergence rate, fecundity (number of eggs per female per batch), and egg hatch rate of *Aedes aegypti.*

Irradiation	Emergence Rate (%)	Fecundity (Number of Eggs per Female per Batch)	Hatch Rate (%)
Control	92.2 ± 1.0 a	72.8 ± 0.3 a	84.3 ± 5.9 a
X-ray of 20 Gy	90.0 ± 1.4 a	72.7 ± 0.3 a	29.0 ± 3.7 b
X-ray of 40 Gy	87.2 ± 1.2 a,b	72.9 ± 0.4 a	5.3 ± 2.6 c
X-ray of 60 Gy	86.1 ± 1.2 a,b	72.5 ± 0.4 a	0.3 ± 0.5 d
γ-ray of 20 Gy	89.4 ± 0.8 a,b	73.0 ± 0.3 a	6.2 ± 0.5 c
γ-ray of 40 Gy	82.8 ± 0.8 b,c	72.7 ± 0.3 a	0.3 ± 0.5 d
γ-ray of 60 Gy	74.4 ± 1.6 c	72.2 ± 0.4 a	0 ± 0 d

The data are shown as mean ± SEM. Values marked with different letters (a–d) indicate significant differences from each other (emergence and hatch rates analyzed using Pearson chi-square and Bonferroni tests; number of eggs assessed by ANOVA and Tukey’s post hoc tests; *p* < 0.05).

**Table 2:** Effects of different doses of X-rays and γ-rays on the average survival time of *Aedes aegypti* males.

Radiation (Gy)	Average Survival Time (X-Rays, Days)	Average Survival Time (γ-Rays, Days)
Control	29.0 ± 0.2 a	29.0 ± 0.2 a
20	27.3 ± 0.2 b	24.7 ± 0.2 c
40	23.4 ± 0.2 c	19.8 ± 0.2 e
60	18.1 ± 0.2 d	14.3 ± 0.1 f

The data are shown as mean ± SEM. Values marked with different letters (a–f) indicate significant differences from each other (Kaplan–Meier analysis and log-rank (Mantel–Cox) test; *p* < 0.05).

**Table 3:** Egg hatch rate of *Aedes aegypti* at different release ratios.

Release Ratio (I:U)	Hatch Rate (X-Rays, %)	Hatch Rate (γ-Rays, %)
0:1	83.3 ± 4.8 a	86.3 ± 4.4 a
1:1	57.8 ± 4.3 b,c	61.2 ± 5.1 b
3:1	37.3 ± 8.6 c,d	42.2 ± 4.5 b,c,d
5:1	29.7 ± 3.4 d	27.3 ± 3.8 d
7:1	24.5 ± 5.2 d	22.5 ± 5.6 d
1:0	1.0 ± 1.3 e	0.3 ± 0.5 e

I: U means irradiated: unirradiated. The data are shown as mean ± SEM. Values marked with different letters (a–e) indicate significant differences from each other (Pearson chi-square and Bonferroni tests; *p* < 0.05). The release ratio of 0:1 was the positive control group.

## Data Availability

The raw data supporting the conclusions of this article will be made available by the authors on request.
